# The role of tactical police medics in the prehospital environment: results from an interview study

**DOI:** 10.1186/s13049-026-01621-5

**Published:** 2026-05-06

**Authors:** Lova Widman, Magnus Andersson Hagiwara, Felix Lindell, Claes Wildar, Denise Bäckström

**Affiliations:** 1 Intern, Norrbotten County Council, Gällivare, Sweden; 2 Centre for Prehospital Research Faculty of Caring Science, Work Life and Social Welfare Boras, University of Borås, Borås, Sweden; 3 Police Education Programme, University of Borås, Borås, Sweden; 4 Department of Biomedical and Clinical Sciences, Linköping University, Linköping, Sweden; 5 Department of Leadership and Command & Control, Swedish Defence University, Karlstad, Sweden

**Keywords:** Tactical medicine, Tactical police unit, Medic training, Prehospital medicine, Interprofessional collaboration

## Abstract

**Background:**

The Swedish tactical police unit is a highly trained team specialized in managing complex situations and tactically demanding operations. Each tactical police group includes a designated medic who completes a 10-week basic training program in tactical medicine, followed by various forms of additional training. The medical aspects of police work are strictly regulated by medical authorities, and among other restrictions, the medics’ authorizations apply only when providing care to fellow officers. The purpose of this study was to explore tactical police medics’ perceptions of their work in relation to training, competencies, tactics, and the provision of practical medical care.

**Method:**

The study employed a qualitative design in which 13 medics from the tactical police unit were interviewed using a semi-structured interview protocol. They were asked questions about their perceived preparedness for the role, their training, and real-life situations in which they had acted as medics. All interviews were digitally recorded and transcribed verbatim. The transcripts were then coded and analysed thematically following Braun and Clarke’s model of thematic analysis.

**Result:**

The results indicated that the medics were generally satisfied with their training and education in tactical medicine. However, there was a clear discrepancy between what they had been trained to do and the tasks they actually performed in their roles. They also expressed frustration about how their mission is defined by medical authorities, particularly the restriction that prevents them from applying their medical authorizations to injured third parties. Collaboration with civilian healthcare was described as inconsistent; cooperation with prehospital physicians was generally viewed positively, whereas the relationship with ambulance personnel was perceived as more challenging.

**Conclusions:**

The tactical police medics represent a highly trained and competent group that is not being utilized to its full potential. They could be deployed more extensively in situations requiring tactical medical care and rescue operations. Achieving this would require stronger and more effective collaboration with civilian healthcare services.

**Supplementary Information:**

The online version contains supplementary material available at 10.1186/s13049-026-01621-5.

## Background

The Swedish Special Intervention Unit (SIU), also known as the national tactical unit, is a specialized police force capable of operating in high-risk and challenging environments [1]. In addition to the SIU, each region in Sweden has a Regional Tactical Unit (RTU) and the three largest cities have a Reinforced Tactical Unit. What distinguishes the Regional Tactical Units from the Reinforced Tactical Units is the training duration and certain capabilities. These units operate in complex and potentially high-risk environments where emergency medical services (EMS) personnel may face threats and violence. The literature highlights that such risks have been documented in a variety of settings, and that efforts to build community relations and preventive work are emphasized to improve safety for all responders [2]. Similarly, there is a risk to the RTU personnel, as exemplified by an American study [3] that underscores the critical importance of timely medical intervention, reporting that 36% of police officers killed in the line of duty might have survived if adequate medical procedures had been administered immediately. Specialist police perform tasks in complex and often demanding environments with heavy equipment, placing them at a high risk of injuries, particularly musculoskeletal injuries, suggesting that personnel in tactical units such as RTUs are similarly exposed to significant physical occupational hazards [4].

The high-risk nature of RTU operations, combined with limited EMS access, underscores the need for advanced prehospital care available near RTU personnel. In 2016, an exception was therefore introduced into Swedish prehospital care regulations. Although the detailed specifications remain confidential, the Swedish Police Journal [5] summarizes the main changes: previously, only authorized healthcare personnel were permitted to administer medications and perform advanced medical procedures in prehospital settings. Under the revised regulation, RTU medics are permitted to provide advanced prehospital care to fellow officers, thereby improving medical support in dangerous environments. However, the updated regulation applies solely to the treatment of colleagues, not civilians. According to the National Board of Health and Welfare, this limitation is based on the rationale that RTU medics are familiar with the physical condition of their colleagues but not that of potential civilian patients [5]. When providing advanced prehospital interventions to individuals other than colleagues, RTU medics must therefore rely on the doctrine of necessity in the Swedish Penal Code [6], which allows legal provisions to be bypassed when necessary to save lives but does not explicitly regulate medical interventions.

Since the regulation was updated, situations that restrict EMS personnel from accessing scenes have increased [2]. The Swedish agency for civil protection and preparedness (MSB) report a rise in incidents involving threats and violence directed at the Emergency Services (EMS, rescue services, and police). Additionally, lethal violence against civilians in Sweden has increased substantially over the past decade with a 50% increase in penetrating trauma, primarily from stab and gunshot wounds [7], and the national terror threat level has been raised from Elevated [3] to High [4] on a five-level scale [8]. Consequently, it may be assumed that the number of dangerous situations requiring prehospital care, both for RTU personnel and for civilians, has also increased.

It remains unknown whether RTU medics have encountered situations requiring them to provide prehospital care to civilians. However, similar specialized police units in neighboring countries have contributed to prehospital care during major incidents involving civilian casualties [9, 10]. Furthermore, prehospital missions conducted by police officers are reported to be beneficial not only in dangerous environments but also when EMS units are unable to reach the scene quickly for example, in connection with cardiac arrest [11, 12] and in traffic accidents [13]. International research has also emphasized the role of police officers in prehospital care which shows that police are many times the first on the scene when responding to traumatic injuries and play an active role through scene management or, in some systems, patient transport [14]. Together, these findings suggest that RTU medics could serve as a complement to EMS in some situations, despite current regulatory limitations. Police officers arriving first at trauma scenes can shorten the time to hospital care by giving an early report and, in certain cases, by transporting the patient to the hospital themselves [15, 16]. Considering this, the National Board of Health and Welfare has called for further investigations into how RTU units (among other Blue Light Agencies) contribute to prehospital care [17].

The current medical training for RTU medics is at least 10 weeks long and has not been scientifically evaluated, and the extent to which their education enhances their medical competence remains unknown. The RTU medical basic training is entirely focused on trauma. During the training, participants learn basic physiology and pathophysiology, as well as assessment and management according to Prehospital Trauma Life support (PHTLS) [18] and Tactical Emergency Casualty Care (TECC) principles [19]. The training includes extensive exercises and practice in simulated environments. In comparison with ambulance personnel in Sweden, the training is generally more extensive, as the ambulance nurse education covers many more areas. Swedish ambulance nurses perceive a great need for more education and training in caring for patients with severe trauma [20]. There is also no comprehensive investigation into the types of medical missions undertaken by RTU medics. Consequently, it remains unclear whether RTU medics consider their training sufficient. Moreover, little is known about how they perceive situations in which they are the only medical resources able to provide prehospital care to civilians. The lack of research in this area also contributes to gaps in the guidelines governing prehospital actions by RTU medics. The absence of such protocols may not only affect patients in need of care but may also place RTU medics in morally challenging situations without adequate support.

The purpose of this study was to explore tactical police medics’ perceptions of their work in relation to training, competencies, tactics, and the provision of practical medical care.

## Method

### Design

This study employed a qualitative interview design using an inductive approach. The interviews were semi-structured and included follow-up in-depth questions, focusing particularly on the participants’ thoughts and experiences. Medics from the Reinforced Regional Tactical Unit in Stockholm, Malmö and Gothenburg were interviewed, as they operate under the same jurisdiction but in different geographical contexts. Thus, the collected material is presumed to reflect the overall situation for Swedish RTU medics.

### Selection and participants

The main selection criterion for the study was being a reinforced regional tactical police officer with medical responsibilities within their unit. Additionally, all participants were required to have worked as RTU medics for more than six months. Participants were initially recruited through purposeful sampling, inviting RTU medics known to the research team and considered likely to provide valuable contacts. Subsequently, snowball sampling was employed to achieve sufficient breadth and depth among participants. This meant that all contacted RTU medics were asked to suggest colleagues who might also meet the inclusion criteria.

In total, 10 individuals were recruited through purposeful sampling and 3 through snowball sampling. All RTU medics who agreed to participate were interviewed, as they met the selection criteria. In total, 13 participants were recruited from three Swedish metropolitan regions: Stockholm, Gothenburg, and Malmö. These regions differ in geographical size, population density, and socio-economic characteristics. Stockholm represents the largest and most densely populated region, whereas Gothenburg and Malmö cover smaller but socio-demographically heterogeneous urban areas. All participants were male, with a mean age of 37.5 years (range: 36–49 years). They completed police academy training between 2010 and 2016 and RTU medical education between 2011 and 2023. Medics from the northern regions did not answer invitations on participations thus limiting conclusion in regard of a geographical difference.

Prior to the interviews, participants received information about the study’s purpose, interview procedures, and data storage. Given the inductive approach, researchers did not share any assumptions about the topic before or during interviews. Operational managers approved participants participation in this study.

#### Previous understandings among authors

The primary interviewers, LW and FL, had no prior knowledge of the subject or its context. Both LW and FL have previous experience in conducting qualitative analysis both through their master thesis using the same methodology, and through two articles not yet published. The author MAH, who conducted some interviews, had experience with qualitative interviews from several previous prehospital studies. He had also prior experience as an RTU medic instructor and was responsible for the RTU medic course. This potential bias was managed by conducting the first interview together with an assistant without such background knowledge and through group discussions of the interview material.

### Collection of data

Initially an interview guide (see Appendix 1) was formed based on the study’s research questions.

- What is the perception of RTU medics regarding how often and in what types of situations they apply their medical skills, and how has this changed over time?
- How do RTU medics perceive the adequacy, relevance, and realism of their medical training in preparing them for real-life prehospital care situations?
- How do RTU medics experience decision-making and manage competing priorities during medical missions, and what are the emotional impacts of providing care during and after missions?

The guide was developed by one of the researchers drawing on experience as an RTU medical instructor (MAH). One pilot interview was performed (later included in the analysis), and minor adjustments were made to the guide following the pilot to optimize coverage of key topics for the semi-structured interviews. The guide primarily served as a foundation for supplementary questions tailored to each participant’s responses. Consequently, data collection was individualized rather than standardized, reflecting participants’ unique experiences.

Prior to the interviews, contact was made with the managers of the RTU units to obtain permission to interview medics. Interviewers LW, MAH and FL communicated with participants via email to establish a professional relationship and ensure trust, as confidential and sensitive topics were to be discussed. All interviews were conducted in Swedish, the native language of both researchers and participants. They were held via video calls to provide flexibility and allow rescheduling at short notice if needed. The interviewers held interviews from offices within Sweden, although the exact location of the participants remained un-known due to secrecy. Interviews were held between fall 2023 and until late spring 2024. The analysis was performed in summer and fall 2024. Answers from participants resulted in both in-depth and open-stated follow-up questions sometimes deviating from the interview-guide, facilitating an inductive collection of data. The usage of an interview guide was mainly as support for the interviewers as a base for further exploration in the areas.

Interviews were recorded with both audio and video, and transcripts were produced either manually by the researchers or using transcription tools, followed by manual review and revision. After the analysis-process, the results were translated to English. The interviews lasted between 42 and 52 min, with a total interview time of 624 min.

### Analysis

**Fig. 1:**
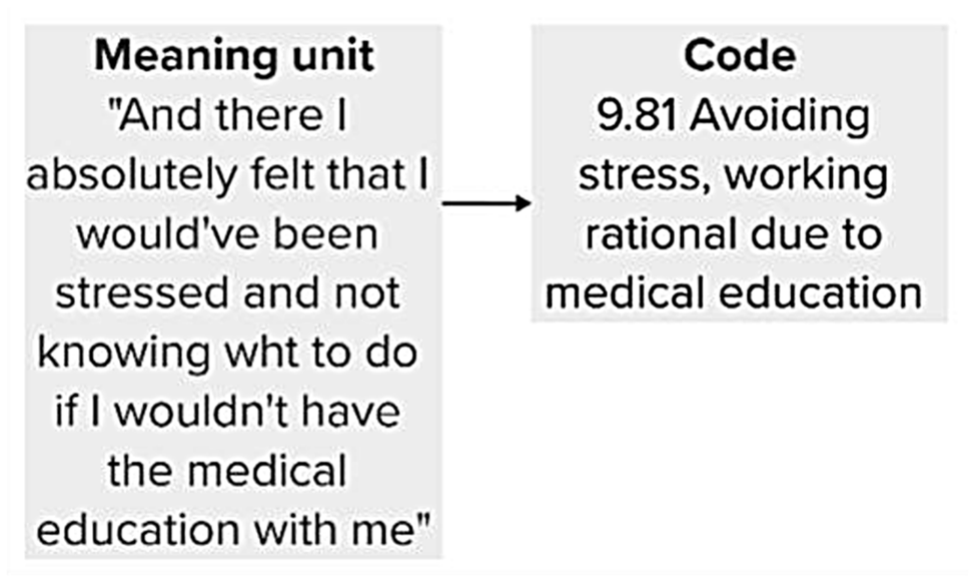
Real example of how a meaning unit from the 9th interview was condensed into a code

The process of analyzing the transcripts followed the approach described by Braun and Clarke [21]. Each transcript was read thoroughly to identify sentences or sections considered particularly relevant in regard of the aim. These segments were marked and defined as meaning units. To manage the large volume of material, each meaning unit was condensed into a code, rewritten in fewer words (Fig. 1). The coding process was reviewed at least twice to ensure sufficient accuracy in rewriting meaning units. In total, the coding process resulted in more than 1,090 codes. Each code was assigned a unique numerical combination to enable traceability back to the original phrase.

**Fig. 2:**
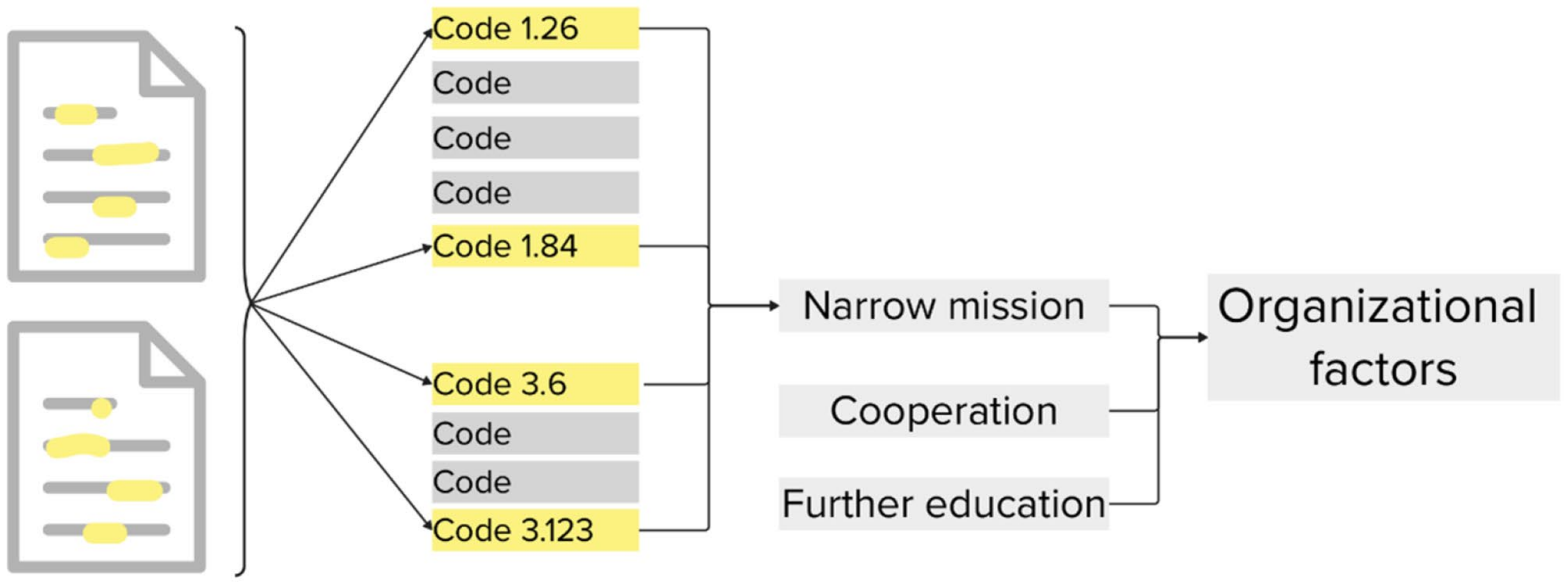
Illustration of the analysis process as described by Braun and Clarke. From left to right, documents are first highlighted with meaningful units, separated as codes. Codes are matched together in regard of common topics, forming sub-themes. Lastly, similar sub-themes are fused together resulting in themes

Codes were transferred to a digital whiteboard and examined individually. Themes were developed by grouping similar sub-themes. Several sub-themes and some themes were later revised as new common patterns emerged (Fig. 2). Thematization/sorting codes into themes were performed independently and subsequently discussed in joint meetings [21, 22].

## Result

**Fig. 3:**
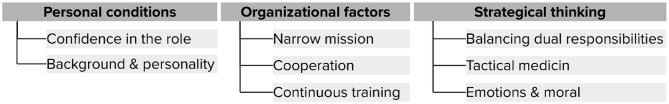
Themes and sub-themes

From the coding process, three themes emerged, describing the fundamental conditions influencing the medics’ role in the prehospital environment (Fig. 3). Personal conditions, organizational factors, and strategic thinking were identified as the foundation shaping RTU medics’ prehospital work and their ability to contribute their expertise. Each theme comprises several sub-themes that further detail the conditions affecting the medics’ work.

### Personal conditions suitable for RTU-medics

Feeling confident in the medical role was considered essential for applying the knowledge acquired through training. This confidence could stem from previous experience, personal attributes, or ongoing training and mental preparedness. Remaining calm and secure in the role was perceived as enabling more rational and automatic actions.

#### Background & personality

Previous experience as response police officers or within the Swedish Armed Forces was considered advantageous for RTU medics, as both backgrounds provided relevant exposure to prehospital situations and injury mechanisms. Several participants reported drawing on experiences from earlier careers when responding to medical missions. Intrinsic motivation, a desire for knowledge, and professional maturity were also identified as qualities that facilitated the translation of theoretical training into effective practice—traits perceived as developing and strengthening over time.

*A strong desire for knowledge characterizes everyone within the tactical unit. Many are not content with just the training they’ve received*,* but have built upon it and become more confident in their roles. (Nr 5)*

#### Confidence in the role

Confidence in the role was regarded as something that could be strengthened both individually and through external support. Training and mental preparedness were highlighted as key factors for feeling secure, as this was perceived strengthening instinctive behaviour and reducing the stress-level. Furthermore, effective teamwork—captured by the notion that a chain is only as strong as its weakest link—was considered to enhance safety. A high level of knowledge was also perceived as facilitating the medic’s work and reducing stress.

*He was shot in the head*,* chest*,* upper arm and leg. And there I felt that I absolutely would’ve been very stressed and wouldn’t known what to do if it wasn’t for the medical education (Nr 9)*

### Organizational factors limiting and facilitating being RTU-medic

RTU medics’ ability to provide medical care was largely attributed to their specialized role, which enabled them to develop deep expertise in trauma care. This narrow focus was considered to support both self-directed learning and effective collaboration. Their capacity to deliver high-quality prehospital care depended not only on organizational factors such as training and supervision but also on the civilian healthcare system’s understanding of the medics’ competencies and limitations.

#### Continuous training

Continuous training was provided both internally and externally. Within the police organization, medics participated in annual medical courses and training sessions, complemented by internships on physician-staffed ambulances and in emergency departments. These internships were regarded as essential for gaining hands-on experience, as internal training sometimes lacked the emotional and situational realism of clinical work. Overall, participants expressed satisfaction with both forms of training and emphasized the value of feedback from civilian healthcare professionals—particularly medically responsible physicians—based on real-life missions.

*It’s a good structure with internships and further education where I look forward to it yearly as it’s there I enhance my skills (Nr 10)*

#### Narrow mission

The RTU medics’ medical mission was described as narrow, limited to the most acute phase of trauma care—often the first 30 min. This focused scope was considered essential, enabling them to maintain high proficiency alongside their duties as tactical police officers. It also facilitated sustained medical training, internships, and ongoing police-related education. Their specialization in early prehospital trauma care was viewed as a valuable complement to civilian EMS, whose broader role requires wide-ranging competence, while RTU medics develop deep expertise within a narrow, time-critical domain.

*In many situations we’re better than ambulances on prehospital trauma-care due to that they need to know so much: they know how to treat kids and elders and everything in-between while we focus on deadly prehospital trauma. Then we become a bit better as we practice that and only think about that (Nr 3)*

#### Cooperation

Cooperation with civilian healthcare—most often ambulance services—was considered essential, as RTU medics either worked alongside ambulance personnel on scene or handed over care for continued prehospital management. However, participants occasionally described unclear responsibilities, since RTU medics possess high competence but hold no formal role within the civilian prehospital system. Collaboration with standard ambulance units was sometimes strained, whereas cooperation with physician-staffed ambulances was described as smooth.

This perception of higher trust and understanding between RTU-medics and physicians, contra the medics and the ambulance-personnel is exemplified by several participants. Most state that this is due to personal knowledge with physicians, as RTU-medics frequently visit the physicians for yearly internships. According to the RTU-medics this result in physicians is more aware of the medics capabilities and limitations, thus facilitating further cooperation. Furthermore, through internship they gain personal knowledge which optimizes work on the field. On the contrary, the ambulance personnel are perceived as lacking on these areas. This both as they do not train with RTU-medics in the way physicians are. Another perspective mentioned by two medics were that the ambulance personnel are greater in numbers, limiting gaining personal knowledge, while the staff-force of physicians in their region is smaller. When working prehospitally, the medics might not meet the same ambulance personnel again.

Participants emphasized that personal familiarity could change collaboration dynamics. One described having lectured for the EMS in his region, which led to increased mutual recognition. Thereafter, cooperation during prehospital procedures improved noticeably, as EMS personnel had a clearer understanding of the RTU medics’ skills and role.

*If more people within the ambulance organization were aware of our capability I believe that our cooperation would be facilitated (Nr 1)*

### Strategical thinking: a key aspect of being RTU-medic

Another key aspect of the RTU medics’ medical missions was their strategic thinking, reflecting principles of tactical medicine that differ from civilian EMS practice. This strategic dimension included knowing when to shift roles, as RTU medics are primarily police officers with police-specific training rather than full-time trauma providers. Their medical responsibilities were therefore integrated into—and continually balanced against—their broader operational duties.

#### Balancing dual responsibilities

RTU medics described their primary role as tactical police officers, with the medical role considered secondary. Beyond providing care, they also educated team members and occasionally acted as medical commanders, guiding stressed ambulance personnel who lacked experience with the injury mechanisms common in RTU operations.

Their medical responsibilities were perceived as extending to civilians, including cases of trauma, cardiac arrest, traffic accidents, and psychiatric emergencies. Treating civilians raised legal and moral dilemmas, particularly when the doctrine of necessity conflicted with formal regulations, creating emotional strain and potentially prolonging care.

Although their medical expertise was highly specialized, their overall role was broad, requiring careful judgment about when to act as medics and when to resume policing duties. This balancing act depended on the strategic conditions of each mission.

*We had a traumatic cardiac arrest*,* knowing that the ambulance was on their way. We wanted to begin treating*,* but at the same time we thought ‘Should we really?’. The ambulance was on its way*,* and she’s almost dead anyways. Should we really begin with our interventions? And there that took me a minute until I took the decision (Nr 5)*

#### Tactical medicine

Participants described tactical medicine as the defining feature of their medical missions, distinguishing their role from civilian EMS. Two tactical factors were considered particularly important: the limited timeframe and the dynamic operational environment. Although many expressed a desire to provide more on-scene care, time constraints often favoured rapid transport, with a “load-and-go” approach deemed more effective in urban settings and “stay-and-play” sometimes necessary in rural areas. The unpredictable and occasionally threatening environment also complicated planning, requiring improvisation and constant prioritization of interventions. These tactical conditions shaped both the scope of care and the overall approach to prehospital management.

*If we weren’t there*,* perhaps nobody would’ve gone in. Even if patrolling officers would’ve gone in*,* although they’re very good*,* they wouldn’t have considered the handing over commenced ‘medical package’ on to the EMS. And there you see our role*,* having a little bit greater knowledge and thinking about integrating everything.* (Nr 3)

#### Emotions & morals

Another aspect of their strategic thinking was recognizing that the medical mission could not always adhere to protocol and sometimes required ad hoc decisions driven by emotional and moral considerations. The restriction limiting advanced procedures to colleagues was frequently cited as an example. Although the medics were aware of these regulations, some described the emotional difficulty of not assisting civilians, particularly in situations where the tactical operation itself might have caused the injuries. As a result, many stated they would rely on the doctrine of necessity when required.

*I see very few limitations when medically treating a civilian being harmed. There’s no one afraid of doing anything due to the consequences*,* people are rather glad to be on site and being able to contribute with one’s knowledge. It happens rather often when we’re out on the field. It’s a traffic accident and you seize the moment to go there to help*,* but also to get experience and to use your knowledge (Nr 9)*

Conversely, many described these ad hoc decisions—particularly those based on the doctrine of necessity—as moral dilemmas. Acting beyond the prescribed scope of practice could potentially lead to distrust from management and, in the worst case, the loss of their medical role. One participant illustrated this dilemma with a traumatic cardiac arrest case: although he possessed the skills to administer fluids and perform advanced airway interventions, the regulations restricted him to providing only CPR. The patient ultimately died.

*If we steps away from the prescript from the National Board of Health and Welfare and if we harm the patient or whatever it will hit us right back when it goes to the Health and Social Care Inspectorate. Perhaps the whole police authority looses the medical ability then? That’s the fear. And even though we can claim the doctrine of necessity*,* it becomes a burden (Nr 5)*

## Discussion

The findings of this study highlight both the strengths and limitations described by medics serving in the Reinforced Regional Tactical Units (RTUs) in Stockholm, Gothenburg, and Malmö. Three overarching themes—personal conditions, organizational factors, and strategic thinking—emerged from the interviews as central to understanding their role in the prehospital environment. These themes illustrate how individual characteristics, organizational structures, and tactical considerations interact to shape the medics’ perceived ability to contribute effectively in high-risk settings. The ability to remain calm under pressure combined with prior experience from the Armed Forces or as response police officers, was described as crucial for effective performance. This aligns with previous descriptions of tactical officers being selected for their capacity to perform under pressure [23]. Research also emphasizes that resilience and stress management are essential traits in prehospital care [24]. Participants’ strong motivation for continuous learning further enhances adaptability in complex environments. These personal conditions suggest that RTU medics are well-prepared to handle morally and emotionally demanding situations, which are otherwise highlighted as particularly challenging in prehospital care [25].

The results illustrate that RTU medics have real-life experience treating civilians; however, many reported being limited in performing advanced medical interventions due to the 2016 regulation. Nevertheless, there are indications that RTU medics can carry out such interventions not only for colleagues but also for civilians. Most importantly, they described personal conditions favourable for these tasks, such as remaining calm under pressure. Previous experience and confidence in one’s role were emphasized not only by participants but also by other healthcare professionals as critical for delivering high-quality care [26]. Prior studies indicate that this particular trait is especially demanding in prehospital contexts, underscoring that RTU medics possess personal conditions advantageous for the role [27, 28].

Secondly, RTU medics described organizational factors as fundamental to the execution of their medical missions. Working within a narrow scope was considered advantageous, as it allowed for specialization in contrast to the ambulance service, which operates under a broad medical mandate. Approximately 15% of all ambulance assignments in Sweden involve traumatic injuries, but only about 5% of these cases are classified as severe, confirming that Swedish ambulance personnel encounter relatively few critically injured trauma patients annually [20, 29]. Nevertheless, cooperation between EMS and police was described as inevitable for several reasons. First, police officers are often first on scene in incidents involving threats and violence. Second, research indicates that police officers have, in some cases, transported patients with penetrating trauma to hospitals without significant differences in mortality rates [16]. Third, participants suggested that their specialization in trauma care could position them as a qualified resource when managing trauma patients. It should be noted that, although RTU medics have appropriate training in trauma care and possess other important skills that make them capable, they do not have the clinical experience of personnel working within EMS. Assessment and decision-making in trauma patients are challenging, and both paramedics [30] and prehospital physicians [31] may have shortcomings in the initial management despite extensive experience. For example, the decision to transport a patient in a police vehicle in order to shorten the time to surgery is complex and requires multiple factors to be considered such as the possibility of performing critical interventions during transport, traffic safety considerations, and the ability to provide early notification to the receiving hospital so that they have time to assemble a trauma team, which often consists of personnel located in different parts of the hospital. At the same time, there are data suggesting that in cases of penetrating injuries, it may be preferable to transport the patient to the hospital by police or private vehicles rather than waiting for an ambulance [32].

Despite their competence, RTU medics’ scope of practice remains narrowly defined, focusing primarily on acute trauma care. While this specialization supports high proficiency, it also creates friction with civilian healthcare providers, particularly ambulance personnel. Cooperation with physician-staffed units was described as smoother, likely due to greater awareness of RTU medics’ training [33]. The interviews show that RTU medics have much closer collaboration with physician-staffed units. Among other things, they often accompany these units to enhance their competence. They do not have the same level of collaboration with standard ambulance services, which leads to a lesser mutual understanding of each other’s competencies. This discrepancy underscores the need for clearer communication and integration between police medics and civilian healthcare systems. Furthermore, reliance on internships and external training highlights the importance of continuous collaboration with civilian healthcare to ensure skill development and mutual trust [34, 35].

The medics’ dual role as police officers and medical providers create unique challenges. Their tactical mindset, balancing rapid evacuation with on-site stabilization, distinguishes their practice from civilian EMS. Tactical medicine was described as fundamentally different due to the dynamic and often threatening environments in which missions occur, requiring improvisation, prioritization, and strategic decision-making under pressure.

However, the restrictive regulation limiting advanced interventions to colleagues’ places medics in moral dilemmas when civilians are injured. When RTU medics are first on the scene where civilians are injured, they are currently unable to fully utilize their skills, such as advanced airway management, pain relief, and fluid therapy. Many participants reported relying on the doctrine of necessity to justify interventions, but this creates uncertainty and fear of repercussions. This tension between legal frameworks and ethical imperatives highlights a critical gap in current policy. The emotional burden of deciding whether to intervene, despite restrictions, was described as significant, with some fearing that deviation from the regulation could jeopardize the entire police medical function.

The study demonstrates that RTU medics often face situations where their expertise could benefit civilians, yet organizational restrictions prevent them from acting fully. This not only risks patient outcomes but also places medics in morally challenging positions. The findings suggest that the current regulation may underutilize a highly trained resource, particularly in contexts where EMS access is delayed or unsafe. This was underscored in the bombings in Oslo 22nd of July 2011 as it is reported that the police were first on scene contributing with first aid for victims [10]. Expanding the scope of RTU medics, while ensuring proper oversight, could lead to improved prehospital trauma care, where the competencies of RTU medics could complement EMS, especially in incidents involving tactical operations and high-risk environments.

Furthermore, threats and violence against EMS personnel have increased in recent years [36], making the presence of RTU medics even more critical in dangerous environments. Their ability to act tactically while providing trauma care positions them as an underutilized resource within Sweden’s prehospital system.

Further research should evaluate the outcomes of RTU medics’ interventions, particularly in civilian cases, to determine their impact on survival and morbidity. Policymakers should consider revising the regulation to allow broader medical authority under defined circumstances, supported by clear guidelines to reduce moral ambiguity. Strengthening cooperation with civilian healthcare through joint training and awareness initiatives could also improve trust and efficiency in prehospital care.

### Limitations

This study has several limitations that should be considered when interpreting the findings. Participants were RTU medics from the largest cities, and while they may operate in diverse geographical settings, the interviews did not provide sufficient detail to draw conclusions about regional differences, limiting transferability [37].

The use of purposive and snowball sampling may have introduced selection bias, as participants with prior connections to the research team or strong engagement in their role may have been more likely to participate. Video-based interviews, while practical, may have constrained interaction and observation of non-verbal cues, and the partly automated transcription process could have introduced minor inaccuracies.

Use of an interview protocol provided structure but may have limited the emergence of additional perspectives; however, follow-up questions were guided by participants’ responses, preserving the inductive approach [37]. Researcher influence is another consideration, as prior experience with RTU medic training could have shaped interview dynamics or analysis, though joint coding and team discussions mitigated potential bias.

Finally, findings are based on self-reported experiences. Concerns about organizational loyalty, potential consequences, or presenting work positively may have influenced responses, particularly regarding ethically complex or procedurally constrained situations, potentially limiting openness or completeness of some accounts.

## Conclusions

This study demonstrates that RTU medics’ prehospital work is shaped by the interplay of personal competence, systemic structures, and tactical demands. Their strong motivation, prior operational experience, and targeted trauma training contribute to effective performance in high-risk environments. However, cooperation with civilian healthcare remains inconsistent, and current regulations limit the medics’ ability to fully utilize their expertise, particularly when civilians are critically injured. These constraints create recurring ethical and operational dilemmas in time-sensitive situations.

Overall, RTU medics constitute a specialized yet underutilized resource within Sweden’s prehospital system. Enhancing collaboration with EMS and clarifying the regulatory framework could strengthen both patient safety and operational effectiveness.

Future research should investigate the clinical outcomes of RTU medical interventions, assess the impact of existing regulatory restrictions, and explore how inter-organizational training can improve cooperation between RTU units and civilian healthcare. Research is also needed on the training and education of RTU medics, as well as on how other prehospital providers perceive collaboration with the group.

## Supplementary Information

Below is the link to the electronic supplementary material.

Supplementary Material 1

## Data Availability

The datasets used and/or analyzed during the current study are available from the corresponding author on reasonable request.

## Abbreviations

CTU: Counter Terrorist Unit
EMS: Emergency Medical Services
SIU: Special Intervention Unit
RTU: Regional Tactical Units

## References

[1] Polismyndigheten Noa. Slutredovisning av regeringsuppdrag att säkerställa insatsförmågan. 2019. https://polisen.se/siteassets/dokument/regeringsuppdrag/slutredovisning-av-regeringsuppdrag-att-sakerstalla-insatsformagan.pdf/download/?v=6b5c050c7a03eb72af6b17340c673d7a. Accessed 8 Apr 2025.

[2] Myndigheten för samhällsskydd och beredskap (MSB). Hot och våld mot blåljuspersonal: en vägledning för ett förebyggande och förberedande arbete med utgångspunkt i social oro. 2022. https://www.msb.se/sv/publikationer/vagledning--hot-och-vald-mot-blaljuspersonal/. Accessed 8 Apr 2025.

[3] Sztajnkrycer MD. Tactical medical skill requirements for law enforcement officers: a 10-year analysis of line-of-duty deaths. Prehosp Disaster Med. 2012;25:346–52. 10.1017/s1049023x00008323 20845323

[4] Lyons K, Pope R, Schram B, Kelly KR, Orr R. Injuries in Specialist Police Officers: A Scoping Review. J Spec Oper Med. 2025;25:62–7. 40513004 10.55460/2OCB-RU71

[5] Wangström J. Vi måste vara självförsörjande på sjukvård. Polistidningen. 2015. https://polistidningen.se/2015/12/vi-maste-vara-sjalvforsorjande-pa-sjukvard/. Accessed 8 Apr 2025.

[6] Swedish Penal Code. (1962:700), Chap. 24, Sects. 1–7. In: Regeringskansliet; 1962.

[7] Holmberg L, Mani K, Linder F, Wanhainen A, Wahlgren CM, Andréasson H. Penetrating trauma on the rise: nine-year trends of severe trauma in Sweden. Eur J Trauma Emerg Surg. 2024;50:3189–97. 39078493 10.1007/s00068-024-02601-z PMC11666756

[8] Swedish Security Service. Raising the terror threat level to high. Säkerhetspolisen. 2023. https://sakerhetspolisen.se/ovriga-sidor/other-languages/english-engelska/press-room/news/news/2023-08-17-terrorist-threat-level-raised-to-high.html. Accessed 8 Apr 2025.

[9] Hansen PM, Mikkelsen S, Alstrøm H, Damm-Hejmdal A, Rehn M, Berlac PA. The Field’s mass shooting: emergency medical services response. Scand J Trauma Resusc Emerg Med. 2023;31:71. 37919753 10.1186/s13049-023-01140-7 PMC10621148

[10] Socialstyrelsen. The bomb attack in Oslo and the shootings at Utøya 2011: KAMEDO report nr. 97. Socialstyrelsen; 2012.

[11] Husain S, Eisenberg M, Police. AED programs: a systematic review and meta-analysis. Resuscitation. 2013;84:1184–91. 23643893 10.1016/j.resuscitation.2013.03.040

[12] Hollenberg J, Svensson L, Rosenqvist M. Out-of-hospital cardiac arrest: 10 years of progress in research and treatment. J Intern Med. 2013;273:572–83. 23480824 10.1111/joim.12064

[13] Lukumay GG, Ndile ML, Outwater AH, Mkoka DA, Padyab M, Saveman BI, et al. Provision of post-crash first aid by traffic police in Dar es Salaam, Tanzania: a cross-sectional survey. BMC Emerg Med. 2018;18. 10.1186/s12873-018-0199-9 PMC6247529 30458715

[14] Salhi RA, Iyengar S, da Silva Bhatia B, Smith GC, Heisler M. How do current police practices impact trauma care in the prehospital setting? A scoping review. J Am Coll Emerg Physicians Open. 2023;4:e12974. 37229183 10.1002/emp2.12974 PMC10204184

[15] Bou Saba G, Bachir R, El Sayed M. Impact of Trauma Center Designation Level on the Survival of Trauma Patients Transported by Police in the United States. Prehosp Emerg Care. 2022;26:582–9. 34550042 10.1080/10903127.2021.1983092

[16] Renberg M, Dahlberg M, Gellerfors M, Rostami A, Günther M, Rostami E. Prehospital transportation of severe penetrating trauma victims in Sweden during the past decade: a police business? Scand. J Trauma Resusc Emerg Med. 2023;31:45. 10.1186/s13049-023-01112-x PMC10492387 37684674

[17] Socialstyrelsen. Sveriges prehospitala akutsjukvård - nulägesbild, bedömning och utvecklingsförslag. 2023. https://www.socialstyrelsen.se. Accessed 8 Apr 2025.

[18] PHTLS. prehospital trauma life support. Burlington, MA: Jones & Bartlett Learning; 2020.

[19] Tactical emergency casualty care (TECC). course manual. Burlington, MA: Jones & Bartlett Learning; 2020.

[20] Abelsson A, Lindwall L. The Prehospital assessment of severe trauma patients` performed by the specialist ambulance nurse in Sweden - a phenomenographic study. Scand J Trauma Resusc Emerg Med. 2012;20:67. 22985478 10.1186/1757-7241-20-67 PMC3509036

[21] Braun V, Clarke V. Using thematic analysis in psychology. Qual Res Psychol. 2006;3:77–101.

[22] Alvinius A, Borglund A, Larsson G. Tematisk analys: din handbok till fascinerande vetenskap. Lund: Studentlitteratur; 2023.

[23] Tedeholm PG, Larsson AC, Sjöberg A. Predictors in the Swedish counterterrorism intervention unit selection process. Scand J Work Organ Psychol. 2023;8.

[24] Hebel K, Jałtuszewska S, Steliga A, Kłosiewicz T, Ślęzak D, Głowiński S. Resilience as a personality trait and stress coping styles: a cross-sectional analysis of a paramedic student cohort. J Clin Med. 2025;14. 10.3390/jcm14061878 PMC11942945 40142686

[25] Bruun H, Milling L, Wittrock D, Mikkelsen S, Huniche L. How prehospital emergency personnel manage ethical challenges: the importance of confidence, trust, and safety. BMC Med Ethics. 2024;25:58. 38762457 10.1186/s12910-024-01061-9 PMC11102201

[26] Jackson BN, Purdy SC, Cooper-Thomas HD. Role of professional confidence in the development of expert allied health professionals: a narrative review. J Allied Health. 2019;48:226–32. 31487363

[27] Jeppesen E, Wiig S. Resilience in a prehospital setting: a new focus for future research? Scand J Trauma Resusc Emerg Med. 2020;28:104. 33087167 10.1186/s13049-020-00803-z PMC7579966

[28] Rosander M, Jonson CO. Professional confidence in the roles as ambulance and medical incident commander. J Conting Crisis Manag. 2017;25:289–300.

[29] Larsson G, Axelsson C, Hagiwara MA, Herlitz J, Magnusson C. Characteristics of a trauma population in an ambulance organisation in Sweden: results from an observational study. Scand J Trauma Resusc Emerg Med. 2023;31:33. 37365663 10.1186/s13049-023-01090-0 PMC10294300

[30] Falk AC, Alm A, Lindström V. Has increased nursing competence in the ambulance services impacted on pre-hospital assessment and interventions in severe traumatic brain-injured patients? Scand. J Trauma Resusc Emerg Med. 2014;22:20. 10.1186/1757-7241-22-20 PMC3994652 24641814

[31] Esmer E, Derst P, Lefering R, Schulz M, Siekmann H, Delank KS. Prehospital assessment of injury type and severity in severely injured patients by emergency physicians: An analysis of the TraumaRegister DGU^®^. Unfallchirurg. 2017;120:409–16. 26757729 10.1007/s00113-015-0127-3

[32] Taghavi S, Chang G, Maher Z, Tatum D, Levy MJ, Raja AS, et al. Mode of transport and prehospital interventions in urban penetrating trauma: A systematic review and practice management guideline from the Eastern Association for the Surgery of Trauma. J Trauma Acute Care Surg. 2026;100:136–46. 41114708 10.1097/TA.0000000000004796

[33] Strandqvist E, Olheden S, Bäckman A, Jörnvall H, Bäckström D. Physician-staffed prehospital units: a retrospective follow-up from an urban area in Scandinavia. Int J Emerg Med. 2023;16(1):43. 37452288 10.1186/s12245-023-00519-8 PMC10349430

[34] Berlin JM, Carlström ED. Why is collaboration minimised at the accident scene? Disaster Prev Manag. 2011;20:159–71.

[35] Berlin JM, Carlström ED. Collaboration exercises—the lack of collaborative benefits. Int J Disaster Risk Sci. 2014;5:192–205.

[36] Stjerna Doohan I, Davidsson M, Danielsson M, Aléx J. Behind the scenes: a qualitative study on threats and violence in emergency medical services. BMC Emerg Med. 2024;24:172. 39322957 10.1186/s12873-024-01090-y PMC11426083

[37] Malterud K. Qualitative research: standards, challenges, and guidelines. Lancet. 2001;358:483–8. 11513933 10.1016/S0140-6736(01)05627-6

[38] World Medical Association. Declaration of Helsinki: ethical principles for medical research involving human subjects. JAMA. 2013;310:2191–4. 24141714 10.1001/jama.2013.281053

