# Supplementary material for: The role of tactical police medics in the prehospital environment: results from an interview study
Source: Scand J Trauma Resusc Emerg Med. 2026 May 6;34:86. doi: 10.1186/s13049-026-01621-5 (PMC13147615; doi:10.1186/s13049-026-01621-5)
Supplement: Supplementary file 1 — Supplementary Material 1 [file 13049_2026_1621_MOESM1_ESM.docx]

**Interview Guide: Police Emergency Medical Care**

**Introduction**

• Information about the purpose of the study

• Do you have any questions about what participation in the study entails?

**Background Questions**

• Age

• Years in the profession; previous profession with medical responsibilities?

• In what year did you complete your emergency medical training?

**Experience**

• How often have you had to act as an emergency medical responder / use your medical skills?

• In what types of situations have you used your medical skills?

• Has the number of situations or the type of situations changed over the years you have worked as a medical responder?

• Have you provided assistance even after ambulance and physician resources arrived?

**Training**

• Do you feel that your training provided you with sufficient knowledge?

• Do you wish you had had more knowledge? If so, in what areas?

• Do we train in a realistic way? Do training exercises differ from real-life situations?

**Task Focus**

• Has it been easy to decide to act as a medical responder?

• Have you experienced a decision conflict where other tasks were required at the same time as your medical skills were needed?

**Emotions**

• How has your experience of acting as a medical responder been?

• Was it difficult/easy to make medical decisions when you were acting in that role?

• Have your feelings during and after an operation as a medical responder changed over the years?

• Have you had the opportunity to discuss your medical decisions afterward?

• Would you have wanted other opportunities for handling the medical aspects after the assignment?

**Conclusion**

• Is there anything that could have made it easier for you when acting as a medical responder?

• Is there anything else we should know about what it is like to act as a police emergency medical responder?
